# Effect of gold addition by the recharge method on silver supported catalysts in the catalytic wet air oxidation (CWAO) of phenol

**DOI:** 10.1039/c9ra00540d

**Published:** 2019-04-09

**Authors:** Adib A. Silahua-Pavón, Gilberto Torres-Torres, Juan Carlos Arévalo-Pérez, Adrián Cervantes-Uribe, Zenaida Guerra-Que, Adrián Cordero-García, Alejandra Espinosa de los Monteros, Jorge N. Beltramini

**Affiliations:** Universidad Juárez Autónoma de Tabasco, Laboratorio de Nanomateriales Catalíticos Aplicados al Desarrollo de Fuentes de Energìa y de Remediaciòn Ambiental, Centro de Investigación de Ciencia y Tecnología Aplicada de Tabasco (CICTAT), DACB Km. 1 Carretera Cunduacán-Jalpa de Méndez AP. 24, C.P. 86690, Cunduacán Tabasco Mexico gilberto.torres@ujat.mx torremensajes@gmail.com +52 19143360928 +52 19143360300; ARC Centre of Excellence for Functional Nanomaterials, The Australian Institute for Bioengineering and Nanotechnology (AIBN) and School of Engineering, The University of Queensland St. Lucia QLD 4072 Australia; Instituto Tecnológico de Villahermosa Km. 3.5 Carretera, Villahermosa – Frontera, Cd. Industrial 86010 Villahermosa Tabasco Mexico

## Abstract

Catalysts Ag/ZrO_2_–CeO_2_ and Au/ZrO_2_–CeO_2_ were synthesized by a deposition–precipitation method and Ag–Au/ZrO_2_–CeO_2_ was prepared using a recharge method for the second metal (Au). The materials were characterized by physisorption of N_2_, XRD, ICP, UV-vis RDS, H2-TPR, XPS and TEM. The results obtained show that the specific areas for monometallic materials were 29–37 m^2^ g^−1^ and 27–74 m^2^ g^−1^ for bimetallics. The tetragonal crystal phase of ZrO_2_ stabilizes when CeO_2_ quantity increases. Using XPS an increment in Ce^3+^ species abundance was determined for bimetallic catalysts in contrast to the monometallic ones; according to the Ag 3d region, this metal oxidation was observed when augmenting the content of CeO_2_ in the materials, and with Au the opposite effect was produced. It was determined by TEM, that the average size of the metallic particles was smaller at bimetallic catalysts due the preparation method. Catalytic activity was evaluated by CWAO of phenol, the Ag–Au/ZrO_2_–CeO_2_ catalyst with 20% wt of cerium reached a degradation of 100% within an hour, being the most active catalyst. Maleic, formic and oxalic acid were identified as reaction intermediates; and at the end of the reaction acetic acid was identified as the main by-product, because it is the most refractory and the conditions for oxidation must be more severe.

## Abstract 2

Addition of gold changed the properties of silver monometallic catalysts by inhibiting the low formation of intermediates and changed of reaction route by formic acid to CO_2_ and water. Furthermore, the bimetallic catalyst showed in the reuse cycles the better stability in CWAO of phenol.

## Graphical abstract

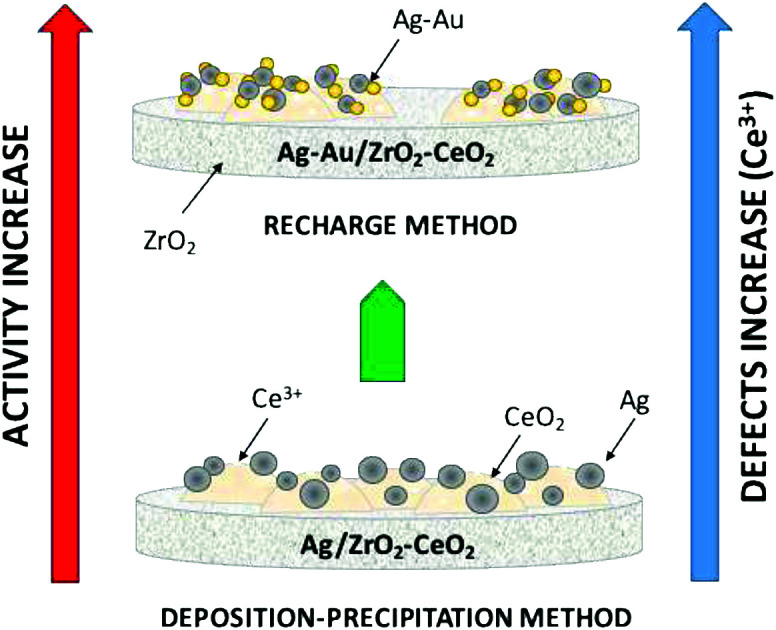

## Introduction

Industrial effluent discharges are considered one of the biggest sources of pollution in water bodies, as they carry organic and inorganic compounds such as: phenols, carboxylic acids, ethers and heavy metals, which cause problems to the environment and human health.^1^ Phenol is one of the most common organic contaminants found in wastewaters, because it is widely used as a precursor for other substances, and for explosives, fertilizers, textiles, adhesives, pharmaceuticals, paper, soap and wood preservatives manufacture. In consequence, residual phenol causes serious damage to human health. In low concentrations, this pollutant is a strong neurotoxin that causes death, affecting vital organs such as kidneys, liver and lungs.^2^ Phenol in aqueous medium has been successfully treated with physical (adsorption),^3^ chemical (oxidation)^4^ and biological (biodegradation)^5^ methods. Among the most efficient chemical methods are the advanced oxidation processes. One of these processes with greater oxidation capacity and volume of treatment is Catalytic Wet Air Oxidation (CWAO), which allows partial or total destruction of organic contaminants in aqueous environment, with the advantage of using an effluent with high concentrations, toxicity decrease and aerobic biodegradability. Mild conditions of both pressure (0.5–10 MPa) and temperature (100–200 °C), and metal catalysts supported on inorganic oxides such as Al_2_O_3_, SiO_2_, TiO_2_, ZrO_2_, CeO_2_ and others^6^ are used in the process to provide positive thermal, mechanical and electronic properties under conditions of pressure and temperature, which ZrO_2_ owns. Also, it is known that the tetragonal phase of zirconia which is thermally stable at high temperatures plays an important role in oxidation reactions due to its high oxygen ion conductivity properties.^7,8^ In addition, the use of ceria increases the oxygen on the surface of the catalyst and improves the oxidation reaction of contaminates. Z. Guerra-Que *et al.*,^9^ reported a study of silver nanoparticles supported on ZrO_2_ modified with Ce to 15 wt% and 20 wt% using CWAO process to destroy the MTBE and they observed a better conversion and selectivity to CO_2_ using catalysts supported on zirconia modified with ceria.

To obtain a higher catalytic efficiency by CWAO, the noble metals (Ir, Pd, Ru, Ag, Au) have been added superficially in supports as active sites, besides they have little leaching capacity in the process in comparison with the metals of transition.^10^ Including these metals are silver and gold. Silver has special characteristics to improve catalytic oxidation reactions. It is known to have a high chemisorption capacity of O_2_.^11–13^ Silver has been studied in the oxidation of CO,^14^ gasoline oxygenates (MTBE),^9^*p*-cresol,^15^ phenol,^16^ among others. On the other hand, gold is catalytically active when dispersed as small particles in an oxide support, the preparation of gold-based catalysts has been widely studied. They are active in many reactions of industrial and environmental importance.^17^ Ngoc Dung Tran *et al.* demonstrated that Au°, is the most active gold species in the Au/CeO_2_ catalysts in the CWAO of carboxylic acids, in addition to showing good yields, attributed by the high mobility of oxygen in the surface of the gold/ceria system.^18^ Also, Au, has been studied in oxidations of volatile organic compounds (VOC such as 2-propanol, toluene and ethanol),^19^ CO,^20^ glucose,^21^ among others. However, it is also reported that gold does not have good stability and its periods of reuse are very short. For this reason, the addition of a second metal can influence the catalytic properties, improving activity, stability and selectivity. To achieve this, it is necessary to have a low reduction element with a high metal support interaction capable of stabilizing and dispersing the first element. In fact, bimetallic combinations such as Au–Ag exhibit significantly improved activity and stability and synergistic effects, reported by Alberto Sandoval *et al.* for oxidations of CO.^22^ This is because Ag has a greater capacity to donate electrons and modifies the electronic properties of gold by a strong interaction between Au and Ag. Another work reported using Ag–Au by photocatalysis. Zielińska-Jurek *et al.* shows that the catalysts of Ag–Au/TiO_2_ obtained a better degradation than the catalysts of Au/TiO_2_ and Ag/TiO_2_ for the degradation of 20 ppm of phenol. However, the problem in photocatalysis is the low concentration of phenol that can be degraded.^23^ Previous work reported Au–Ag/ZrO_2_–CeO_2_ catalysts synthesized by the redox method for MTBE oxidation where the bimetallic catalysts showed better activity than the catalysts Ag/ZrO_2_CeO_2_, on the other, the catalytic activity of Au–Ag/ZrO_2_–CeO_2_ is also shown synthesized by deposition–precipitation with urea for the oxidation of phenol by CWAO, obtaining a conversion of 61% and a TOC of 40%.^24^

The synthesis of bimetallic Ag–Au catalysts has been reported by different methods, such as dealloying for borohydride electro-oxidation,^25^ microemulsion,^26^ sequential deposition–precipitation with urea,^24^ KOH^19^ and NaOH,^22^ and redox.^27^ The latter offers the advantage of performing a selective deposition of a metal on another metal's surface, both in reduced state in presence of hydrogen. Electron configurations, the atomic radio of metals and low temperatures of preparation are variables that affect the synthesis of bimetallic catalysts. In this case, the second metal can be dispersed as a monolayer on the surface of a first metal forming solid solutions with comparable particle sizes.

Considering the above, in this paper bimetallic catalysts were prepared using a mixed oxide ZrO_2_–CeO_2_ as support, by sol–gel method varying the content of the second oxide. The first metal (Ag) was added by the deposition–precipitation method using NaOH as the dispersing agent, then the second metal (Au) was incorporated by the Recharge method (redox) using a 1 : 1 molar ratio between Ag and Au.

## Results and discussion

### (a) Elemental analysis

The theoretical metal loadings of the monometallic and bimetallic catalysts were 1.4 wt% for Ag and 2.5 wt% for Au, which corresponds to molar ratio 1 : 1 (Ag : Au) for the bimetallics. Table 1 compares the theoretical and the measured gold and silver loadings in wt% and the Au/Ag atomic ratios for the Au–Ag samples. As expected for AgZr and AgZrCe, practically all the silver present in solution was deposited on the catalysts. In the case of AgZr, AgZrCe10 and AgZrCe20, about 93, 85, 93% was deposited on the catalysts respectively. The Au actual deposited on the monometallic were 88, 92 and 92% for 0, 10 and 20% w/w cerium respectively. These results showed that the deposit–precipitation method produces a good behavior for the deposit of Ag and Au on the simple and mixed oxide surface. In the case of bimetallic samples, the actual gold loadings are also close to the theoretical value (2.5 wt%), whereas for silver, the actual loading is always lower than the nominal loading. According with the molar ratio of AgAu was found the ratios between 0.91 and 0.95 that is very close to the theoretical ratio.

**Table 1:** Theoretical and actual Au and Ag loadings measured by ICP analysis for the mono and bimetallic catalysts

Catalyst	Metal loading (%)				Actual Ag/Au molar ratio
	Theoretical		Actual		
	Ag	Au	Ag	Au	
AgZr	1.4	—	1.3	—	—
AgZrCe10	1.4	—	1.2	—	—
AgZrCe20	1.4	—	1.3	—	—
AuZr		2.5		2.2	
AuZrCe10		2.5		2.3	
Au		2.5		2.3	
AgAuZr	1.4	2.5	1.2	2.4	0.91
AgAuZrCe10	1.4	2.5	1.2	2.3	0.95
AgAuZrCe20	1.4	2.5	1.2	2.3	0.95

### (b) N_2_ physisorption

**Fig. 1:**
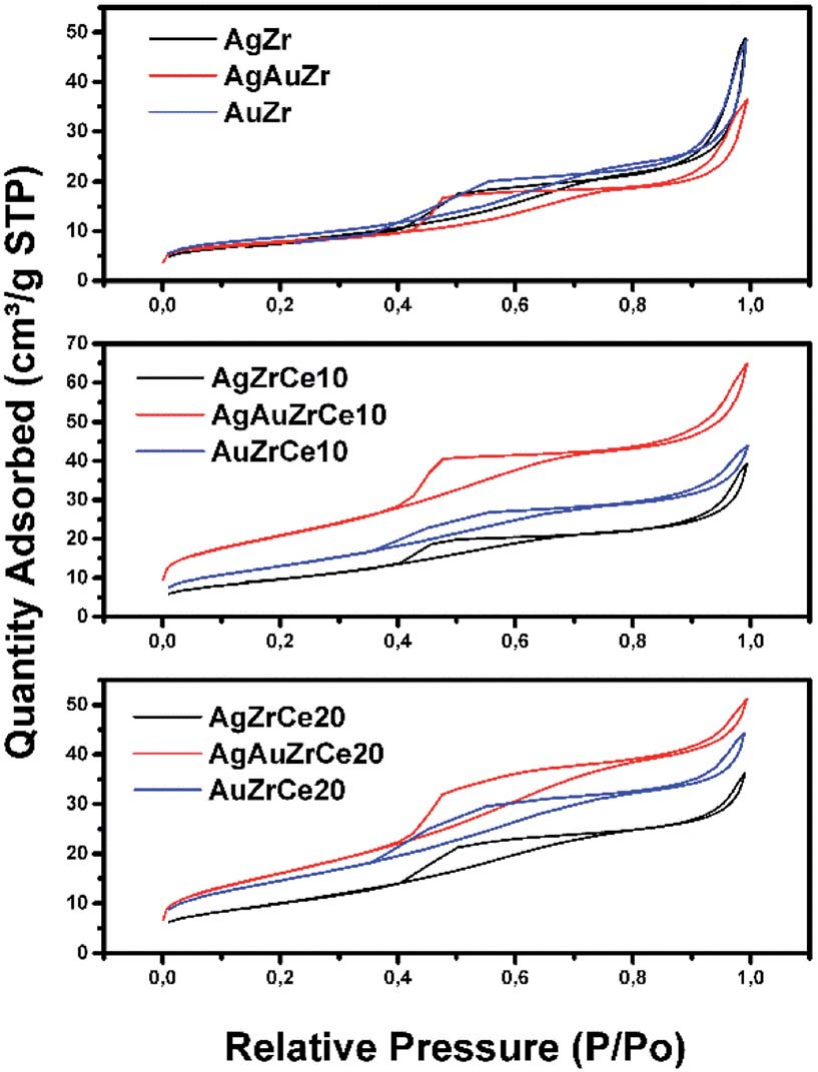
Nitrogen adsorption isotherms of Ag, Au and Au–Ag catalysts on ZrCe with 0, 10 and 20 wt% in Ce.

**Fig. 2:**
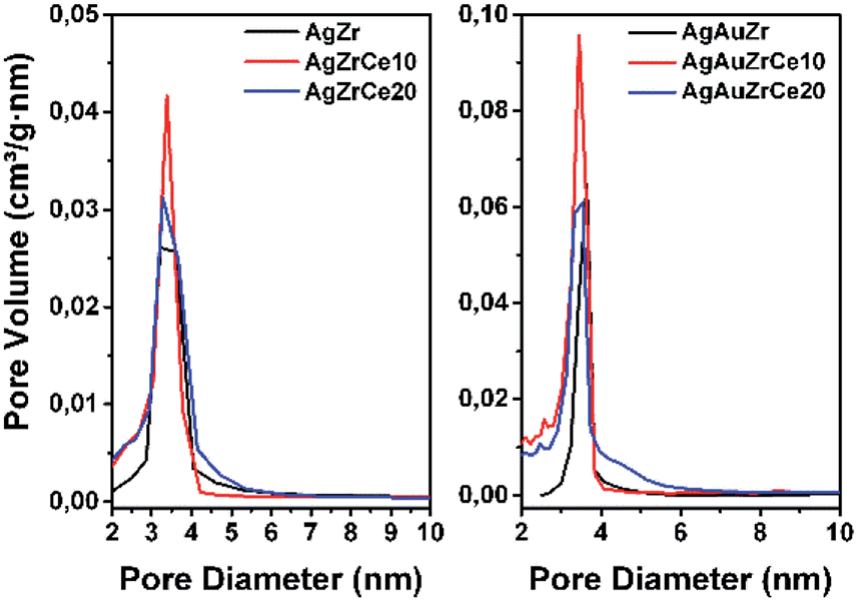
Pore distribution for monometallic and bimetallic catalysts.

All samples show (Fig. 1) a type IV isotherm (IUPAC classification) indicating that the catalysts are mesoporous. The hysteresis loop (H2 type) is commonly associated with the presence of ink-bottle shaped pores, which could be due to inter- or intra-particle porosity.^28,29^ As also observed, hysteresis indicates the presence of capillary condensation suggesting the presence of high-strength agglomerates (aggregates).^30^ On the other hand, it is observed in Fig. 2, the pore distribution of the monometallic and bimetallic catalysts. All the samples presented a pore distribution of the unimodal type.

Table 2 reports the calculated surface areas. Regarding the surface area an increase was observed when the concentration of cerium oxide increases. Ag monometallic catalysts obtained areas of 29 to 37 m^2^ g^−1^ and the Au catalysts showed areas of 32 to 53 m^2^ g^−1^; these phenomenon's is attributed to the difference between ions of Zr^4+^ (0.084 nm) and Ce^4+^ (0.098 nm), causing Ce ions not being introduced into the ZrO_2_ structure; remaining on the surface of the material and therefore increasing the specific surface area.^31^ In addition, the surface areas are a little more in the Au than the Ag monometallic, for effect of the metal particles size. Meanwhile specific area between 27 and 74 m^2^ g^−1^ is found on bimetallic catalysts; in comparison with the monometallic catalysts, an increase in the area of about 2 times can be observed in the bimetallic catalysts containing 10 and 20% in cerium. This effect could probably be due to the acid conditions (pH = 1) of synthesis of the second metal, resulting in the redispersion of the Ce and unblocking pores on the surface of the catalyst. This effect is not observed with AgAuZr.

**Table 2:** Surface characteristics and crystal size of monometallic and bimetallic catalysts

Catalyst	*S*_A_ (m^2^ g^−1^)	Pore size (nm)	Pore volume (cm^3^ g^−1^)	Crystal size^a^ (nm)	Metal particle size^b^ (nm)
AgZr	29	7.9	0.077	8.8	10
AgZrCe10	35	5.4	0.059	7.7	6.6
AgZrCe20	37	4.7	0.058	7.5	6.1
AuZr	32	6.5	0.061	8.7	4.5
AuZrCe10	48	6.1	0.061	8.1	4.3
AuZrCe20	53	5.6	0.064	7.9	3.9
AgAuZr	27	6.8	0.057	8.9	6.8
AgAuZrCe10	74	4.8	0.1	8.9	5.7
AgAuZrCe20	60	4.4	0.079	7.6	4.2

a Crystal size by Scherrer's equation (nm).

b Metal particle size by TEM.

### (c) X-ray diffraction

**Fig. 3:**
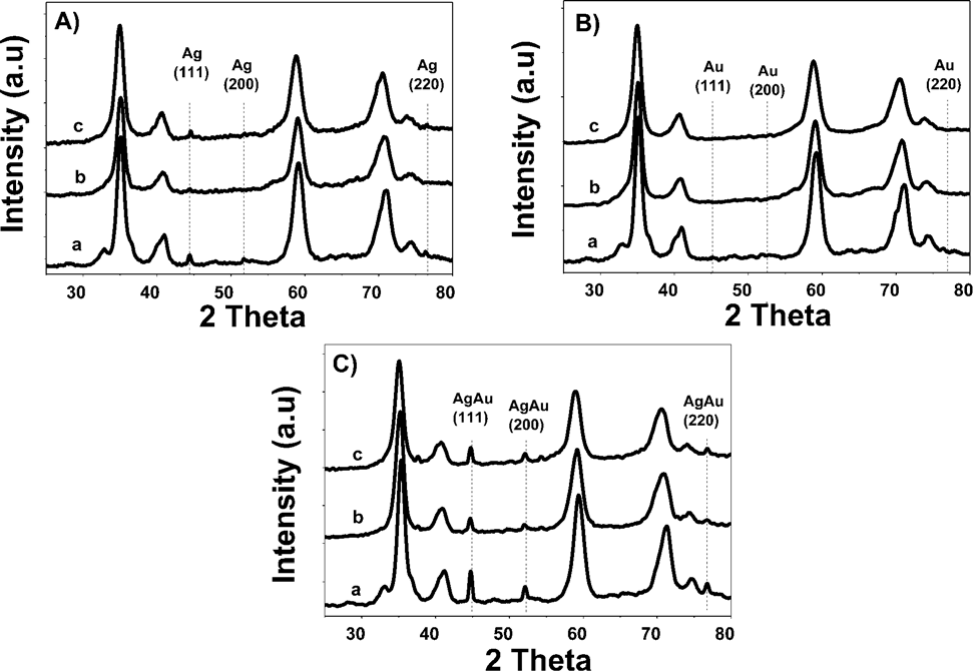
X-ray patterns of monometallic (A) Ag and (B) Au and Au–Ag bimetallic (C) catalysts, where a: Zr, b: ZrCe10 and c: ZrCe20.

Fig. 3 shows diffraction patterns of monometallic and bimetallic catalysts.

Phases corresponding to monoclinic and tetragonal crystal structures of zirconium oxide were identified. This, two structures coexist in the AgZr, AuZr and AuAZr catalysts with the following diffraction patterns (Fig. 3A): (a) tetragonal phase on angles 35.18, 40.03, 40.97, 59.12, 69.96, 70.14 and 74.40;^32^ corresponding to planes (101), (002), (110), (200), (103), (211) and (202) and (b) monoclinic phase at 32.81° of 2*θ* of the plane (111),^33^ and it is observed higher abundance of tetragonal phase. However, in catalysts with high concentrations of cerium (10–20%), only the tetragonal crystalline phase could be observed, therefore, when increasing the amount of CeO_2_, the material tends to stabilize to the tetragonal phase. In these synthesized catalysts it was not possible to observe the presence of the cubic phase (cerianite) characteristic of cerium oxide, this result agrees with that reported by E. Rubio-Rosas *et al.*^34^ The Ag monometallic showed a weak peak corresponding to silver phases around at 2*θ* = 44, 52 and 77. In addition, the intensity of peaks decreases when increase the cerium amount in the catalyst. In the Au monometallic Fig. 3B, the diffraction peaks corresponding to Au phase were not detected. It can be attributed to the small size of gold particles, indicating the high dispersion of Au *via* the precipitation–deposition technique, this effect showed by E. Hernández-Ramírez *et al.*,^35^ with 3 wt% over TiO_2_ using the same method. Concerning the bimetallic catalysts, the identified structures were tetragonal and monoclinic of zirconium oxide. Angles 38.60 and 55.73 corresponding to the planes (200) and (220) characteristic of cerium oxide cubic structure were also identified; only on catalysts with higher concentration of cerium. Recharge method re-dispersed the cerium deposited on the surface to agglomerate it in larger crystals, see Fig. 3C. As for the deposited noble metal, three diffractions were identified, characteristic of the cubic phase centred on the silver faces, at 2*θ* = 44.44, 51.95 and 76.49 corresponding to planes (111), (200) and (220).^36^ The diffractions characteristics of gold are similar to those of silver; this is because both metals have the same crystalline phase (fcc). Just an increase in the diffraction intensity was observed due to the presence of the second metal, regarding monometallic catalysts. The bimetallic catalysts AgAuZrCe10 and AgAuZrCe20 presented a lower intensity to the metallic phases in contrast to AgAuZr, this could be due to a better dispersion of gold in the catalysts with cerium. Table 2 presents the calculation of the average size of the zirconium oxide crystal for catalysts (monometallic and bimetallic), which was determined by FWHM (full width half maximum) using the Debye–Scherer equation.^37^ The average crystal size of ZrO_2_ in all synthesized materials was 7.5–8.9 nm. Materials with smaller average crystal size were the catalysts with 20% wt of cerium; Ag, Au monometallic and Au–Ag bimetallic with 7.5, 7.9 and 7.6 nm respectively.

### (d) UV-vis spectroscopy

**Fig. 4:**
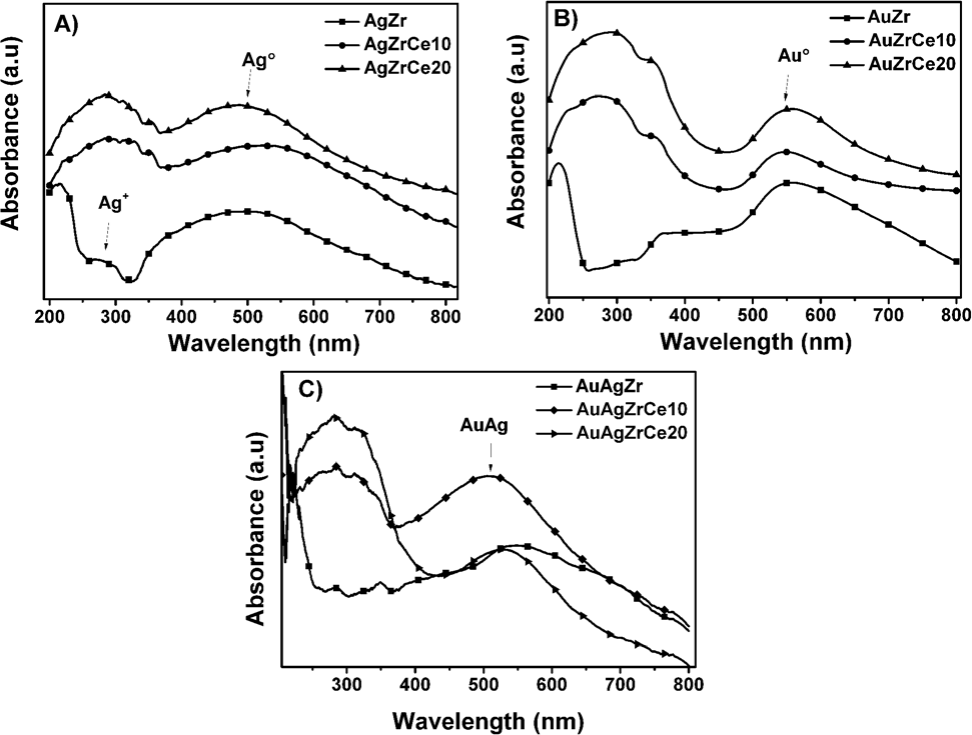
UV-vis spectra of monometallic silver (A) and gold (B) and bimetallic silver–gold (C) catalysts.

Fig. 4A shows spectra of monometallic catalysts. Absorption bands around wavelength maximum between 450 and 500 nm were observed, related with the presence of silver metal nanoparticles. These bands are due to the resonance absorption of the surface plasmon.^38–40^ It has been reported that bands between 230 and 260 are attributed to ions Ag^+^ y Ag^+^_*n*_ related to highly dispersed silver. Furthermore, the plasmons centered between 290 and 350 are assigned to agglomerations of metallic silver Ag^0^_*n*_.^41^ These absorptions are only clear in AgZr. An increase in the range of 250–320 nm can be observed at spectra corresponding to the catalysts with cerium. Cerium oxide is an n-type semiconductor with a prohibitive energy band (bandgap) of 3.1 eV.^42^ It is known to have a strong absorbance in the ultraviolet range where two types of characteristic bands to 250 and 297 nm can be shown, these assigned to the transfers of charges Ce^3+^ ← O^2−^ and Ce^4+^ ← O^2−^ respectively.^43^ In relation of gold, it is reported that the characteristic bands of clusters are of 280 and 300 nm in materials with Zr/Ce > 1 and gold metal plasmon (Au°) is observed between 500 and 600 nm bands^7^ Au catalysts showed (Fig. 4B) in the all samples a plasmon around 550 nm, corresponding to Au°. Another hand, the Au–Ag bimetallic spectra (Fig. 4C) there is a change on the surface resonance plasmon in contrast of monometallic, moving towards the visible one; this is caused by the incorporation of Au, which is in higher concentration than Ag.

### (e) Temperature programmed reduction (H_2_-TPR)

**Fig. 5:**
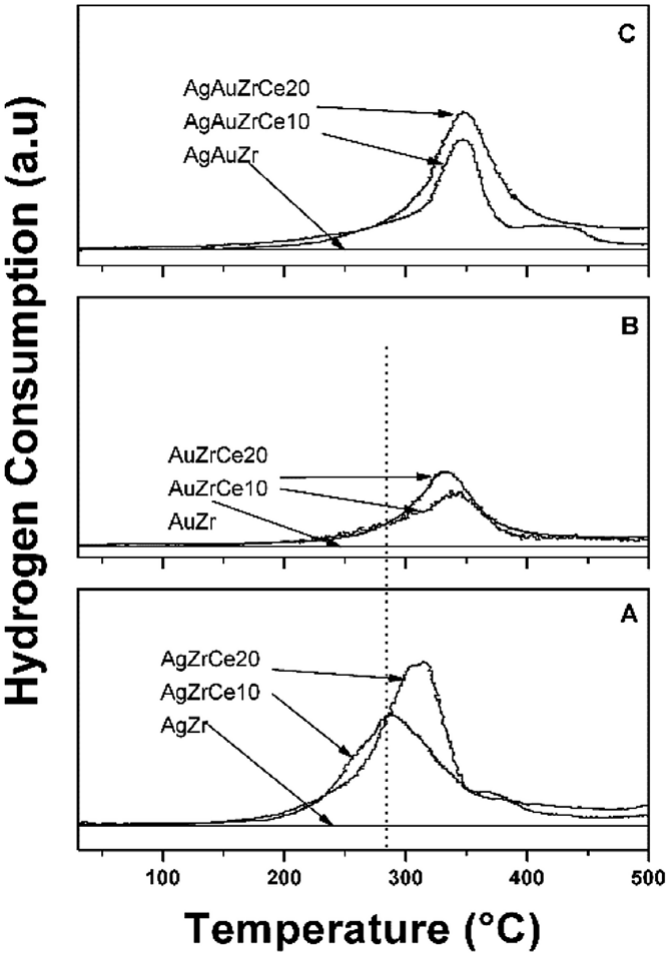
TPR profiles of catalysts: (A) Ag, (B) Au monometallic and (C) AuAg bimetallic.

The results of the treatment at programmed temperature are shown in Fig. 5. The reduction profile for the AgZr sample did not show hydrogen consumption in a range of analysis. However, samples with cerium, AgZrCe10 and AgZrCe20, showed a signal close to 300 °C, related to the reduction of silver oxide and another related to cerium oxide (≈370 °C); the difference between samples lies in the amount of hydrogen consumed that goes according to the amount of cerium in the samples. For gold monometallic, the AuZr sample showed no hydrogen consumption. With respect to the samples with cerium, only the reduction of the cerium oxide present was observed. The sample AgAuZr did not show reduction in the analysis performed. The reduction of cerium oxide and silver oxide was displaced at higher temperatures compared to the silver monometallic, the interaction between the metals on the surface, modify the temperature. In the three cases analyzed, the AgZr, AuZr and AgAuZr catalysts did not show hydrogen consumption, the analysis was carried out after the respective thermal treatments.

### (f) X-ray photoelectron spectroscopy (XPS)

**Fig. 6:**
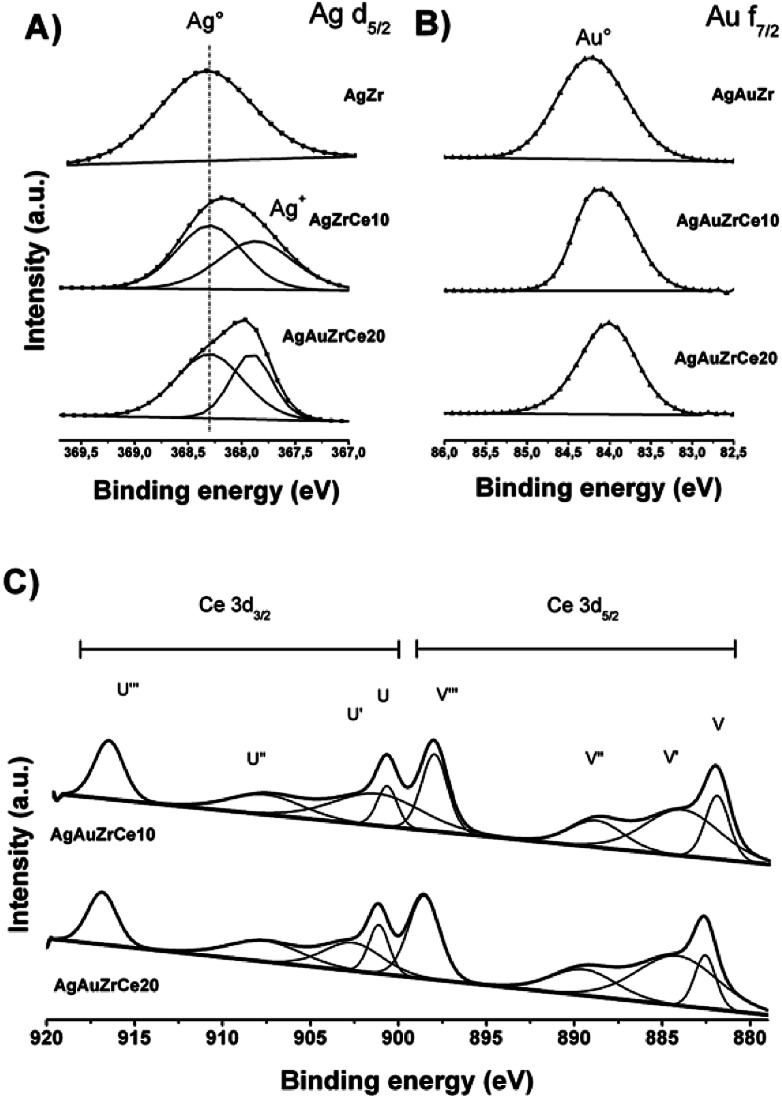
Silver XPS spectra in bimetallic catalyst (A), gold in bimetallic catalyst (B) and cerium in monometallic and bimetallic catalysts (C).

Fig. 6A shows de-convoluted XPS spectra for level Ag 3d_5/2_ of monometallic catalysts. Two picks are reported for level 3d_5/2_ of silver: 368.3 eV characteristic of metallic species Ag° and 367.99–367.8 eV for the oxidized species Ag^+^.^44–46^ For AgZr only the peak at 368.3 eV was observed, this indicates that the deposited silver is totally reduced. For catalysts with high concentrations of cerium (10–20%), a mixture of Ag° and Ag^+^ states coexists on 368.3 and between 367.97–367.88 eV respectively. Table 3 reports the relative abundances obtained. This effect in the oxidation states of Ag, on the monometallic catalysts modified with Ce, may be due to a strong electronic interaction of Ag^0^ with Ce^4+^, causing the partial oxidation of Ag^0^ to Ag^+^and the reduction of Ce^4+^ to Ce^3+^(Table 3).

**Table 3:** Oxidation states and surface atomic ratio of monometallic and bimetallic catalysts

Catalysts	Oxidation states							Surface atomic ratio			
	Zr^4+^ (3d_5/2_)	Ce % (3d_5/2_)		Ag % (3d_5/2_)		Au% (4f_7/2_)					
		Ce^4+^	Ce^3+^	Ag^0^	Ag^+^	Au^0^	Au^+^	O/Ce	O/Ag	O/(Au + Ag)	Au/Ag
AgZr	182.08	—	—	100	—	—	—	—	9.48	—	—
AgZrCe10	182.06	70	30	77	23	—	—	0.83	7.68	—	—
AgZrCe20	181.96	69	31	62	38	—	—	0.91	8.49	—	—
AuZr	182.15	—	—			100	—	—		—	—
AuZrCe10	182.03	67	33			100	—	0.81		—	—
AuZrCe20	181.89	66	34			100	—	0.77		—	—
AgAuZr	182.33	—	—	100	—	100	—	—	—	13.74	0.80
AgAuZrCe10	182.19	59	41	54	46	100	—	0.67	—	7.25	0.90
AgAuZrCe20	181.26	57	43	50	50	100	—	0.61	—	6.93	0.92

On the other hand, the monometallic gold catalyst showed an abundance of 100% for all the samples for the Au metallic species. This result is corroborated by H2-TPR (Fig. 5), where no signals are observed after the reduction treatment. In the case of Zr analyzed by XPS, a signal about 182 ± 0.3 eV was found, characteristic of the oxidation state Zr^4+^,^47^ in the case of the synthesized silver monometallic catalysts, binding energies between 181.96 and 182.08 eV were found, the monometallic Au catalysts showed an effect similar that the silver catalysts with binding energy signals between 182.15 and 181.89 eV, proving that there was no change of state when incorporating cerium, gold and silver. Regarding cerium oxide, the Ce 3d spectra show a great variety of peaks, these are found in two energy levels which are Ce 3d_3/2_ and Ce 3d_5/2_, where the two oxidation states are found; Ce^4+^ with characteristic peaks V, V′′, V′′′, U, U′′ and U′′′, Ce^3+^ with V′ and U′, as seen in Fig. 6C. Presence of Ce^3+^ is frequently observed in mixed oxide systems of ZrO_2_–CeO_2_ and TiO_2_–CeO_2_.^48–50^ However, for relative abundance calculation only the de-convolved areas of level Ce 3d_5/2_ were analysed as reported by some authors.^51,52^Table 3 reports the relative abundances obtained. In the bimetallic catalysts it is observed that when the second metal is deposited (Au), the percentage of oxidized silver increases (Ag^+^), this is observed in AgAuZrCe10 and AgAuZrCe20, which implies that there is an interaction between silver and gold. In the work of Zanela *et al.*, it is mentioned that the redox potential Au^3+/^Au^0^ is greater than that of Ag^+^/Ag^0^; As a consequence, the gold precursor (HAuCl_4_) is capable of oxidizing silver metals.^22^ In addition, the reduced silver status is maintained in the AgAuZr catalyst.

Fig. 6B shows the binding energies for the Au 4f_7/2_ level of the bimetallic catalysts. In the case of gold, the energies in 84 eV represent Au metallic and species between 85.6–85.7 eV for the oxidized species Au^+^.^53–55^ In our work we find signals at 84.0, 84.1 and 84.2 for AgArZr, AgAuZrCe10 and AgAuZrCe20. Casaletto *et al.* They analyzed the Au nanoparticles in different metal oxide supports and found two gold states, Au^0^ and Au^+^. The state Au^+^ was shifted by *Δ* = 2 eV from the Au^0^ state.^56^ In our case, it moved up to *Δ* = 0.2 eV. For this reason, it's can affirm that gold is in a reduced state. The little displacement may be due to a strong interaction between Au and Ce.

Spectra of Ce 3d_5/2_ (Fig. 6C) for bimetallic catalysts indicated the presence of two oxidation states Ce^3+^ and Ce^4+^, in the same way than in monometallic catalysts, showing abundance of 59 and 57% for the state Ce^4+^ and 41 and 43% for Ce^3+^ for catalysts with AgAuCe10 and AgAuCe20 respectively, this indicates that there is a greater abundance of Ce^3+^ cations on the surface in contrast to the monometallic ones. The presence of the reduced species Ce^3+^ are associated with generation of oxygen vacancies because of charge compensation.^48,49^ As in other investigations, crystalline defects, such as oxygen vacancies may occur in the tetragonal structures of the ZrO_2_–CeO_2_ systems, with a particle size smaller than 15 nm.^57–59^

Regarding the surface oxygen ratio, monometallic catalysts showed an increase due to the presence of cerium (O/Ce). Part of the oxygen because of the contribution of cerium, and a percentage associated with oxidized silver (O/Ag). The bimetallic catalysts synthesized by recharge method favoured the formation of vacancies in the cerium oxide, this can be observed in the O/Ce ratio, as well as the reduction of gold (O/Ag + Au). The real Ag : Au atomic ratio was found between 0.8–0.92 close to the theoretical ratio proposed to the synthesis of these materials.

### (g) TEM (transmission electron microscopy) and HRTEM (high resolution transmission electron microscopy)

**Fig. 7:**
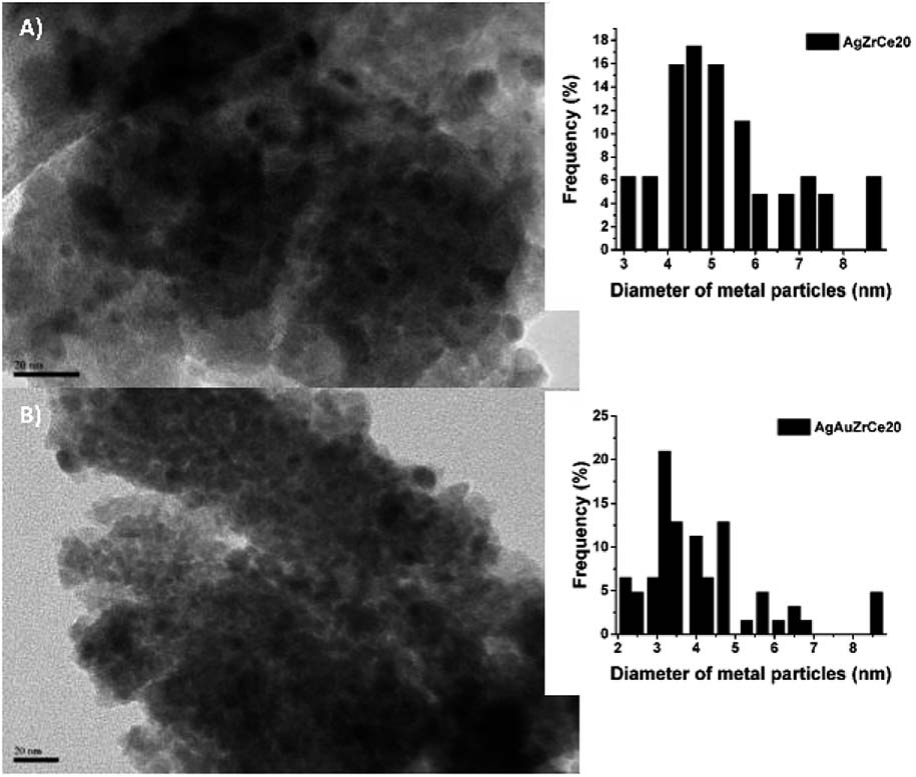
TEM images and distribution of monometallic and bimetallic catalysts particles.

Based on the TEM generated images, the average metal particle size of the monometallic and bimetallic catalysts was determined; Table 2 shows these results. The particle size distribution for the silver monometallic catalyst, shows 17% of particles larger than 8 nm; the highest concentration is between 4–6 nm and 45%, see Fig. 7A. After deposition of the second metal, a re-dispersion of the basic metal (Ag) was observed, causing the silver particles, smaller than 8 nm, to decrease in size; the monometallic catalysts of Au obtained an average metal size of 4.5, 4.3 and 3.9 for AuZr, AuZrCe10 and AuZrCe20 respectively, the gold catalysts showed the lowest average particle size in comparison with the other materials synthesized in this work. The result can be beneficial in the reaction of oxidation of phenol, it is known that the size of gold particles is of fundamental importance to obtain better results in oxidation reactions.^60^ The highest concentration of metal particles (Ag–Au) was centered between 3–5 nm with a percentage of 64%. In the bimetals, 9% of metal particles were observed between 2–3 nm, probably these are contributed by gold, because in the Ag monometallic catalysts the smallest particle quantified was 3 nm, see Fig. 6B.

**Fig. 8:**
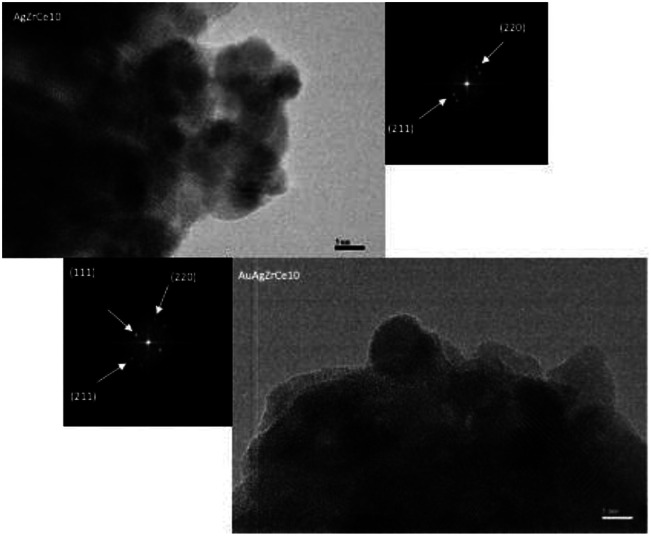
HRTEM images and distribution of monometallic and bimetallic catalysts particles.

The HRTEM image of the AgZrCe10 sample can be seen in Fig. 8. The measurement of the interplanar distances was made and it was identified in the plane (211) of the tetragonal zirconia. It is also identical to the plane (220), corresponding to the ceramic oxide in its cubic structure. For the sample AuAgZrCe10, three planes (211), (220) and (111) were identified; Corresponding to tetragonal zirconia, ceramic oxide and silver oxide respectively. A difference in the results by X-ray diffraction, by HRTEM was possible to identify the presence of cerium oxide and silver oxide. These two species are found in the respective samples with a size that cannot be observed by X-rays. In the case of Au and Ag metallic can't be discriminated, having a very similar interplanar distance (Ag (111) d (A) = 2.359 and Au (111) *d* = 2.355).

### (h) Catalytic activity

**Fig. 9:**
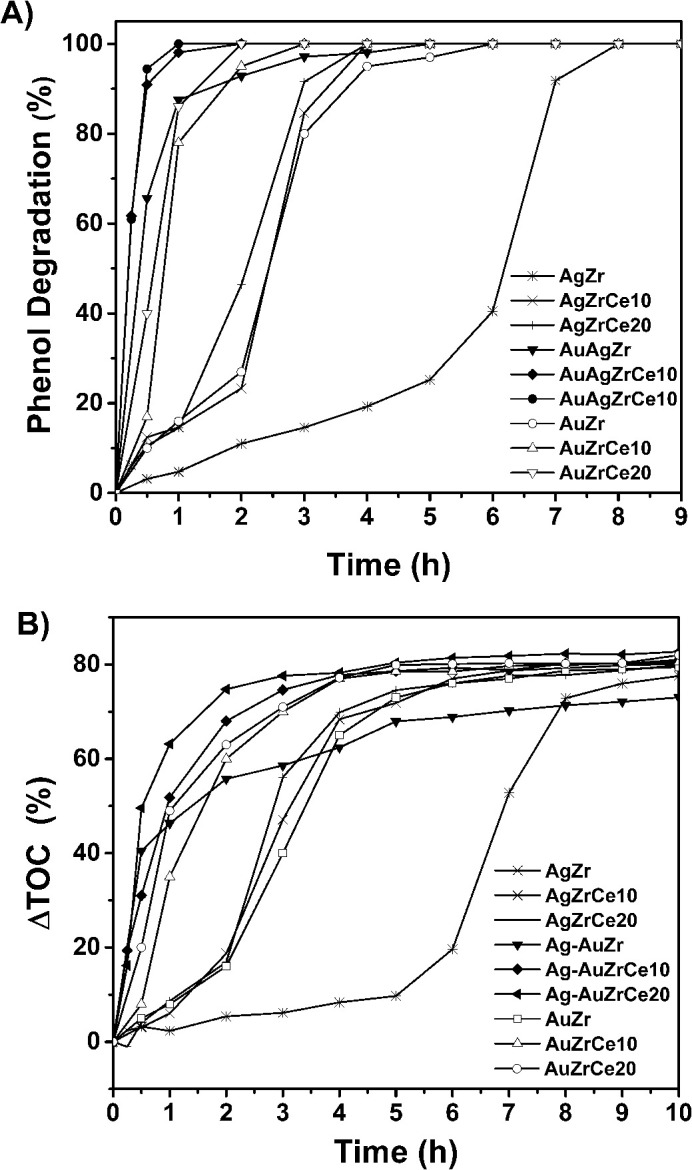
Phenol degradation in terms of time (A), total organic carbon abatement in terms of time (B).

Fig. 9A shows the results of the catalytic study for the oxidation of phenol using the catalysts AgZrCe and AgAuZrCe. A great difference can be observed between the Ag and Au monometallic catalysts. where 100% conversions were obtained at 7, 4 and 4 h for the AgZr and AgZrCe10 and AgZrCe20 catalysts respectively and the AuZr, AuZrCe10 and AuZrCe20 at 5, 3 and 2 h respectively, where the gold monometallic showed a better result that the ag monometallic clearly. However, the best effect to decrease the time for the phenol conversion were using the bimetallic catalysts for AgAuZr, AgAuZrCe10 and AgAuZrCe20 with 4, 2 and 1 hour respectively. According to the reaction rate (Table 4), the AgAu catalysts showed around 10 times faster than the monometallic catalysts, where the catalyst with the highest reaction rate was AgAuZrCe20 with 21.2 mmol h^−1^ g^−1^. Fig. 9B shows the behaviour to % ΔTOC. Table 4 shows the results obtained at one hour of reaction. AgAuZrCe catalysts showed the best performance to % ΔTOC with 51.8 and 63.1 for 10 and 20% in cerium respectively. Instead, Ag monometallic showed a low abatement between 2.3–6.0% and Au monometallic between 8.0–49.5%.

**Table 4:** Conversion, TOC, selectivity to CO_2_, rate and TOF of monometallic and bimetallic catalysts in CWAO of phenol

Catalyst	*X*_phenol_ (%)	ΔTOC (%)	*S*_CO_2__ (%)	*r*_A_ (mmol h^−1^ g^−1^)	TOF (h^−1^)
	1 h	1h	1 h		
AgZr	4.7	2.3	49.1	0.5	4.1
AgZrCe10	14.7	6.0	41.1	2.6	21.1
AgZrCe20	14.5	8.7	59.9	2.3	19.1
AuZr	16.1	8.0	49.7	1.7	12.8
AuZrCe10	78.3	35.4	45.2	8.3	62.7
AuZrCe20	86.2	49.5	57.2	9.1	68.9
AgAuZr	87.5	46.3	52.9	10.2	43.1
AgAuZrCe10	98.1	51.8	52.8	20.7	90.1
AgAuZrCe20	100	63.1	63.2	21.2	92.1

The action of cerium in the reaction is evident by producing a lower concentration in TOC abatement. This phenomenon can be explained according to the information of Table 3's data, a greater amount of Ce^3+^ helps to promote oxidation; this is due to the facility of the cerium oxide to pass from Ce^4+^ to Ce^3+^ state and its action as a source of oxygen for the phenol molecule oxidation.^61^ The turn over frequency. The turnover frequencies (TOFs) were examined for the conversion of phenol to CO_2_ as function of the Ag, Au and AgAu metal on catalyst ZrCe. TOF analysis is shown in Table 4. The AgAu catalysts showed a higher TOF compared to the Ag and Au catalysts with 43.1, 90.1 and 92.1 h^−1^ for AgAuZr, AgAuZrCe10 and AgAuZrCe20 respectively. While Ag and Au catalyst between 4.1–21.1 and 12.8–68.9 (h^−1^) respectively. This may be due to a saturation in the active sites due to the presence of acetic acid as an intermediate in the reaction, preventing the interaction of free phenol with the Ag sites. This is corroborated with the study of the selectivity of the reaction intermediates.

#### (h.1) Intermediates study

**Fig. 10:**
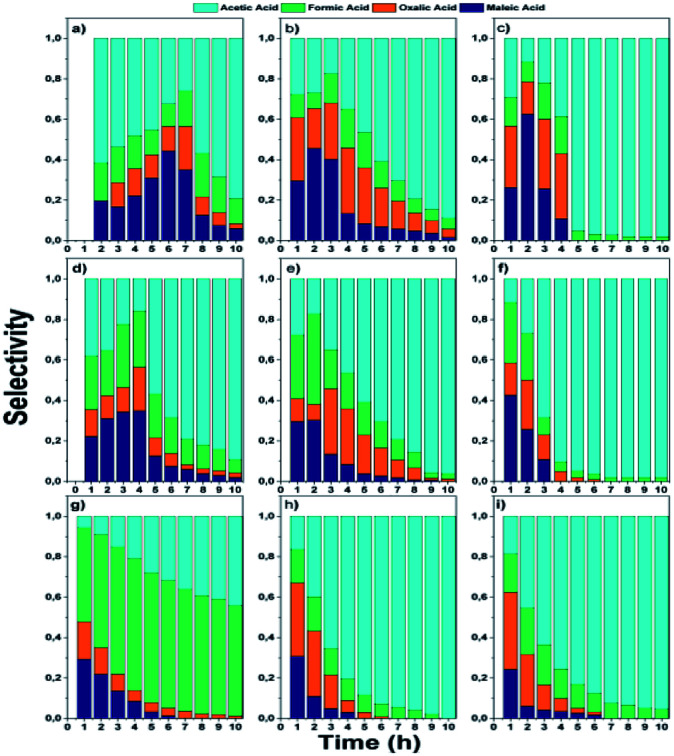
Selectivity to intermediaries from phenol oxidation: monometallic; (a) AgZr, (b) AgZrCe10, (c) AgZrCe20, (d) AuZr, (e) AuZrCe10, (f) AuZrCe20 and bimetallic catalysts (g) AgAuZr, (h) AgAuZrCe10 and (i) AgAuZrCe20.

The intermediaries identified were formic, acetic, oxalic and maleic acid. The selectivity to intermediaries from CWAO of phenol is observed in Fig. 10.

**Fig. 11:**
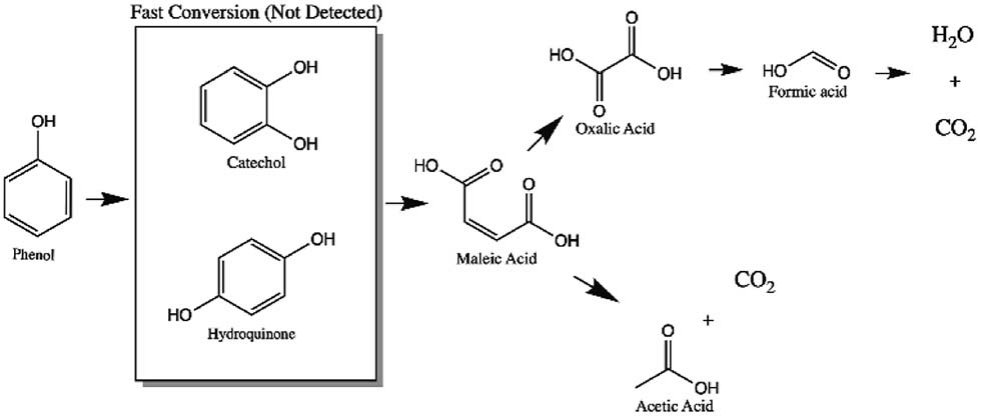
Route of the reaction proposed by CWAO of phenol using mono and bimetallic catalysts.

In the monometallic catalysts the identified intermediates were maleic, acetic, formic and oxalic acid. The AgZr catalyst showed a very important selectivity for acetic acid in the first 6 hours of reaction and (Fig. 10a), however, according to the selectivity behaviour, the AgZr catalyst took a long time to convert the acetic acid to CO_2_. Thus, some of the active sites were saturated, causing a very low reaction rate and conversion, shown in Fig. 9 and Table 4. Meanwhile, the AgZrCe catalysts with 10 and 20% in ceria showed similar behaviour to AgZr, but in a shorter time of 2 and 1 hour of reaction, accelerating the oxidation process of acetic acid (Fig. 10b and c). This is caused by the amount of cerium that contributes a greater quantity of oxygen to the surface of the catalyst, benefiting the oxidation of the intermediaries. For the monometallic gold catalysts (Fig. 10d–f), a greater preference can be observed towards the intermediates, oxalic acid and formic acid in comparison with the monometallic silver catalysts, this means that the gold catalysts have a greater preference towards the reaction route for maleic acid–oxalic acid–formic acid–CO_2_. In addition, it can be observed that acetic acid is a final product in the reaction. In addition, it can be observed that acetic acid is a final product in the reaction. On the other hand, the AgAuZr catalyst (Fig. 10 g) showed a greater selectivity to formic acid, since a greater selectivity was observed in comparison to the other intermediates. That is, gold provides another reaction route different from AgZr, besides significantly reducing the number of intermediaries with respect to silver at the end of the reaction, where only acetic and formic acid are observed. In Fig. 10h and 9i, the behaviour of the catalysts AgAuZrCe10 and AgAuZrCe20 respectively is observed. In comparison with AgAuZr, a high selectivity to formic acid is not observed, this is caused by the rapid selectivity of formic acid to CO_2_. This is proven by the increase in CO_2_ selectivity (Table 4). In addition, it is possible to observe a greater selectivity to oxalic acid than to maleic acid. It is reported by other authors that the route of oxidation of phenol by oxalic acid and formic acid is easier to reach CO_2_ and H_2_O than the route by acetic acid. In all catalysts the production of acetic acid was identified, which did not degrade; it is reported that for the degradation of the latter, higher reaction temperatures are required; around 200 °C,^62^ for this reason acetic acid could not be degraded since the reaction conditions were not the optimum, being the by-product of the reaction. After the analysis of the selectivity of intermediaries for the oxidation of phenol. In Fig. 11, the reaction route for the monometallic and bimetallic catalysts is proposed.

**Fig. 12:**
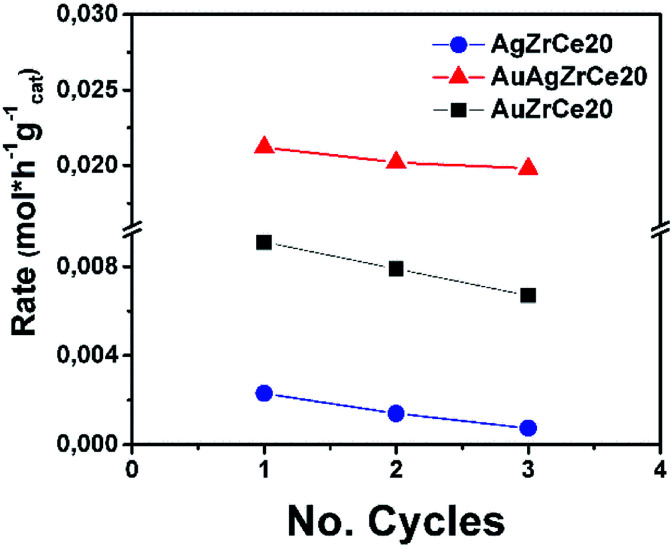
Reuse cycles for AgZrCe20 and bimetallic AgAuZrCe20 catalysts in CWAO of phenol.

It is of extreme importance to explore the stability and recyclability of the catalyst material as it could reduce the cost of the catalytic process and therefore results in promising catalyst. In the Fig. 11 showed the reuse cycles of the better catalyst in CWAO of phenol in this work. Moreover, AgAuZrCe20, AgZrCe20 and AuZrCe20 were compared to study the stability of the material. The reaction was carried out at 160 °C and 8 bar of O_2_, the reaction rate was quantified at the end of one hour of reaction. In each recycle, the catalyst was filtered and washed with methanol several times and the solvent were evaporated at a temperature of 120 °C, finally it was given a heat treatment with H_2_ at 400 °C for 1 hour. Fig. 12 show the results obtained. In the case of the AgZrCe20 catalyst it showed after of the third reuse a loss of reaction rate about 70% and AuZrCe20 with 22%. Meanwhile, the bimetallic catalyst showed 10% only. In such a way, the interaction gold/silver provides an improvement in stability and reusability in phenol oxidation has been showed clearly.

## Experimental

### (a) Supports synthesis

Zirconium oxide synthesis (ZrO_2_) was prepared by zirconium butoxide hydrolysis (Sigma-Aldrich, (Zr(OC_4_H_9_)_4_) 80% wt on 1-butanol), using butanol (Aldrich 98%) as solvent medium. The alkoxide/water ratio was 1 : 16 and the water/alcohol ratio was 1 : 8 molar. Water/butanol mixture was added in a three-necked flask, heated in recirculation at 70 °C. At the desired temperature, the zirconium butoxide was slowly incorporated for 3 hours. The pH was adjusted to 3 with acetic acid (J. T. Baker 98%) and left in recirculation for 24 hours. Once the gel was formed, it was subjected to the extraction of liquids in a rotary evaporator (Büchi-R-II) at a temperature of 60 °C.

ZrO_2_–CeO_2_ supports were synthesized using the same methodology as for ZrO_2_, first dissolving cerium precursor in a water–butanol mixture; which was cerium acetylacetonate (Sigma-Aldrich, Ce(C_5_H_7_O_2_)_3_·*x*H_2_O). Subsequently the zirconium butoxide was slowly added. All materials were exposed to an oxidizing atmosphere with a heating ramp of 2 °C min^−1^ until reaching 500 °C to stabilize the phases. The %wt of cerium was 10 and 20.

### (b) Ag monometallic catalysts synthesis

The silver deposit was carried out by deposition–precipitation method, starting from silver nitrate (J. T Baker, (AgNO_3_), 99.7%), which was dissolved and stirred with oxide immersed in the solution for 20 minutes. Later, the temperature was warmed until 80 °C, and a solution of NaOH (J. T Baker, (NaOH) 98.1%) was slowly added, with a concentration of 0.5 M reaching a pH of 11. The mixture was left stirring for 2 hours. The sample was centrifuged to separate the phases, the solid was recovered and washed with distilled water at 50 °C, the washing process was repeated until a pH of 7 of the leached water was obtained. The material was dried at 120 °C for 12 hours. The product was oxidized in an oxidizing atmosphere, and then reduced in a reducing atmosphere (H_2_). Both thermal treatments were completed on a heating ramp of 2 °C min^−1^ and a gas flow of 60 mL min^−1^; final temperature was 300 °C for oxidation and 400 °C for metal reduction. The symbology of these materials is AgZr and AgZrCe*X*; where *X* is the percentage of cerium added.

### (c) Au monometallic catalyst synthesis

The preparation of Au monometallic sample with 2.5 wt% Au as theoretical loading was performed by deposition–precipitation with urea (DPU) in the absence of light. Briefly, the amount necessary of gold precursor, HAuCl_4_, and the urea (0.42 M) were dissolved in 100 mL of distilled water. Then, the support was added to this solution; thereafter, the suspension temperature was increased to 80 °C and kept constant for 16 h under stirring. After, the material was dried at 120 °C for 12 hours. Finally, the thermal treatments of the gold were similar that Ag catalyst. The symbology of these materials is AuZr and AuZrCe*X*; where *X* is the percentage of cerium added.

### (d) Bimetallic catalysts synthesis

Monometallic silver catalysts were modified by gold addition carried out with the recharge method reported by author J. Barbier *et al.*^63^ The molar ratio of the second metal (Au) was 1 : 1 regarding the present metal (Ag); using tetrachloroauric acid as precursor (Sigma-Aldrich, (HAuCl·6H2O), 99.999%).

The monometallic catalyst was introduced in a glass reactor, subsequently hydrogen was introduced by bubbling (1 h) and a further nitrogen treatment (0.5 h), allows elimination of dissolved or reversibly adsorbed hydrogen. The gold solution (HAuCI_4_) was introduced as an acid solution in which HCl was added to adjust the pH to 1. So freshly reduced catalyst was maintained in gold solution in the reactor and continuously stirred by a counter-current nitrogen flow (1 h). After the reaction, bimetallic catalyst prepared was dried until room temperature, then left at a temperature of 120 °C and a heating ramp of 2°C min^−1^ for 12 h. Finally, the catalyst was activated by hydrogen at a temperature of 400 °C for 4 h. The symbology for these materials is AgAuZr and AgAuZrCeX; where *X* is the percentage of cerium added.

### (e) Characterization

#### (e.1) ICP-analysis

Chemical analysis of Au and Ag in the dried samples was performed by ICP in a Varian Vista Pro ICPOES instrument. The Au and Ag weight loadings were expressed in grams of each metal per gram of sample.

#### (e.2) BET method (specific area, diameter and pore volume)

The determination of the specific area, diameter and pore volume of the catalysts was realized by N_2_ physisorption technique using BET method. It was performed in an equipment of surface area and porosimetry systems measurement, MICROMERITICS TRISTAR 3020 II brand, at 77 K (−196 °C). A 0.2 g sample was weighed and degassed during 2 hours at 300 °C to remove impurities.

#### (e.3) X-ray diffraction

Planes, crystalline phases and crystal size were identified with this characterization, an X-ray diffractometer, Rigaku Miniflex brand, was used, with a radiation source Co Kα, at *λ* = 1.790307 Å; 30 kV and 15 mA. The average crystallite size of oxide catalyst was estimated using the Scherrer equation:
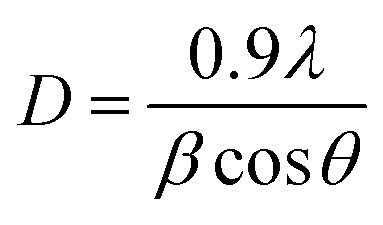


#### (e.4) UV-vis RDS spectrophotometer

Presence of the metal and the possible oxidation states were determined with this technique. The characterization was performed on a UV-vis spectrophotometer, Varian brand model Cary 300, which has a range of 900 to 190 nm, with diffuse reflectance accessory (coupled integration sphere). The BaSO_4_ compound was used as a reference with 100% reflectivity.

#### (e.5) Transmission electron microscopy (TEM)

It was performed on a JEOL JEM2100 STEM. Equipped with an energy dispersive X ray analyser (JEOL JED 2300) (EDX). Samples were ground and suspended in ethanol at room temperature and dispersed with stirring in an ultrasonic bath for 15 min, later, a solution aliquot was deposited through a copper grid to determine the metallic particles size on catalysts.

#### (e.6) Temperature-programmed reduction (H_2_-TPR)

The programmed temperature reduction was carried out in a BELCAT Basic B instrument with a sample amount of 0.2 g, using the following methodology: degassing in a nitrogen atmosphere at a temperature of 300 °C for 30 minutes with a flow of 10 mL min^−1^. Subsequently, it was allowed to cool to room temperature, the gas was changed to a mixture of hydrogen–nitrogen (5%/95%). The temperature rose at a rate of 10 °C min^−1^ until reaching 500 °C.

#### (e.7) X-ray photoelectron spectroscopy (XPS)

Oxidation states and relative abundances were determined by the results obtained from a KRATOS Axis ULTRA X-ray photoelectron spectrometer, incorporated with a hemispherical analyser of electron energy of 165 mm. The incident radiation used was monochromatic Al Kα X-rays (1486.6 eV) at 225 W (15 kV, 15 mA). The pressure in the analysis chamber of samples was 1 × 10^−8^ torr. Data was analysed by XPS Casa software, version 2.3.14, and using as a reference carbon at 285.0 eV to adjust the peaks of the species studied.

### (f) Catalytic evaluation (catalytic wet air oxidation)

Wet air oxidation of phenol was performed in a stainless-steel Parr reactor with a capacity of 600 mL. The reaction was carried out at 160 °C and a partial pressure of 8 bar oxygen (ultra high purity 99.9999%) as oxidizing agent. Stirring of 1000 rpm during the reaction. A solution of 350 mL with a concentration of 1000 ppm and a catalyst ratio of 1 g L^−1^ with respect to the aqueous solution were introduced. Samples were taken at different times.

#### (f.1) High performance liquid chromatography (HPLC)

A Shimadzu Prominence HPLC Chromatograph was used with a Bio-Rad model Aminex HPX-87 fabricated of stainless steel and with the following measures: 300 mm height and 7.8 mm internal diameter, with a refractive index detector (RID-10A). The conversion of phenol and the selectivity of the intermediate was determined with the following equations:^64^
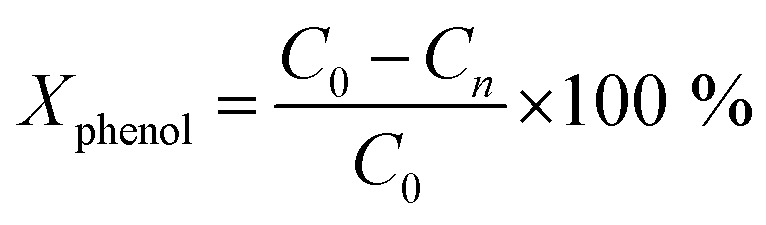

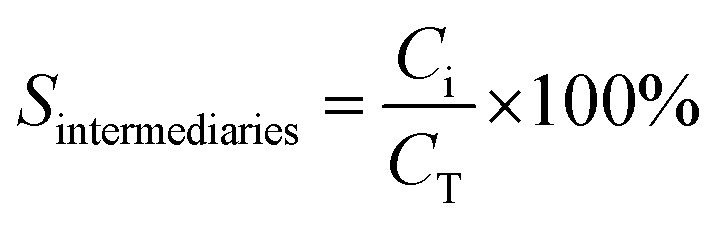
where *C*_0_ is a initial concentration of phenol and *C*_*n*_ is the phenol concentration to different reaction times. *C*_i_ and *C*_T_ are the amount of one intermediate formed and the total amount of the other intermediates at a given time respectively. The initial rate (*r*_i_) was calculated from the phenol concentration as a function of time, using the follow equation:
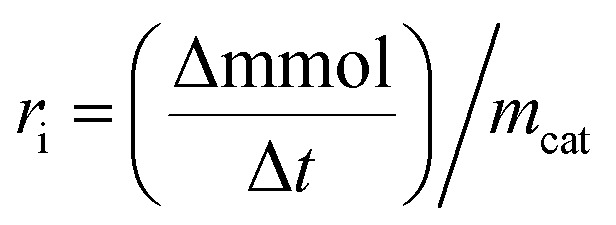
where 
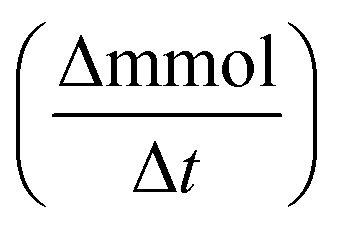
 is a phenol concentration and *m*_cat_ is the mass of the catalyst.

#### (f.2) Total organic carbon (TOC)

A Shimadzu TOC-VCHS analyser was used to obtain this parameter, employing a nondispersive infrared detector to quantitatively analyse carbon dioxide originated by the sample. The % ΔTOC was calculate with the equation:
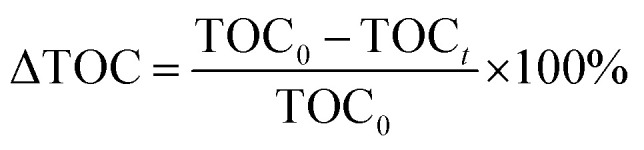
where TOC_0_ is total organic carbon at *t* = 0 and TOC_*t*_ is total organic carbon at different time in the oxidation reaction. The selectivity to CO_2_ was calculated according to follow equation:^65^
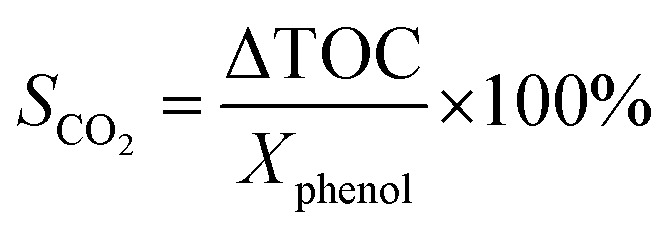


## Conclusions

Strong interaction between added superficially metals (Ag–Au) with 1 : 1 molar ratio was found, due to the method of preparation. The prepared materials were evaluated by CWAO of phenol, in catalytic tests it was revealed that the addition of Au decreased the time of degradation. A significant effect was observed with the presence of CeO_2_ in the materials, which led to a lower formation of reaction intermediates and auto-oxidation of Ag and auto-reduction of Au. The presence of reduced Ce^3+^ species is associated with the generation of oxygen vacancies due to the charge de-compensation on the surface atoms of the catalyst. The addition of Ce caused an important TOC reduction in phenol CWAO, because of the oxide-reduction capacity of Ce^4+^/Ce^3+^.

**Fig. 13:**
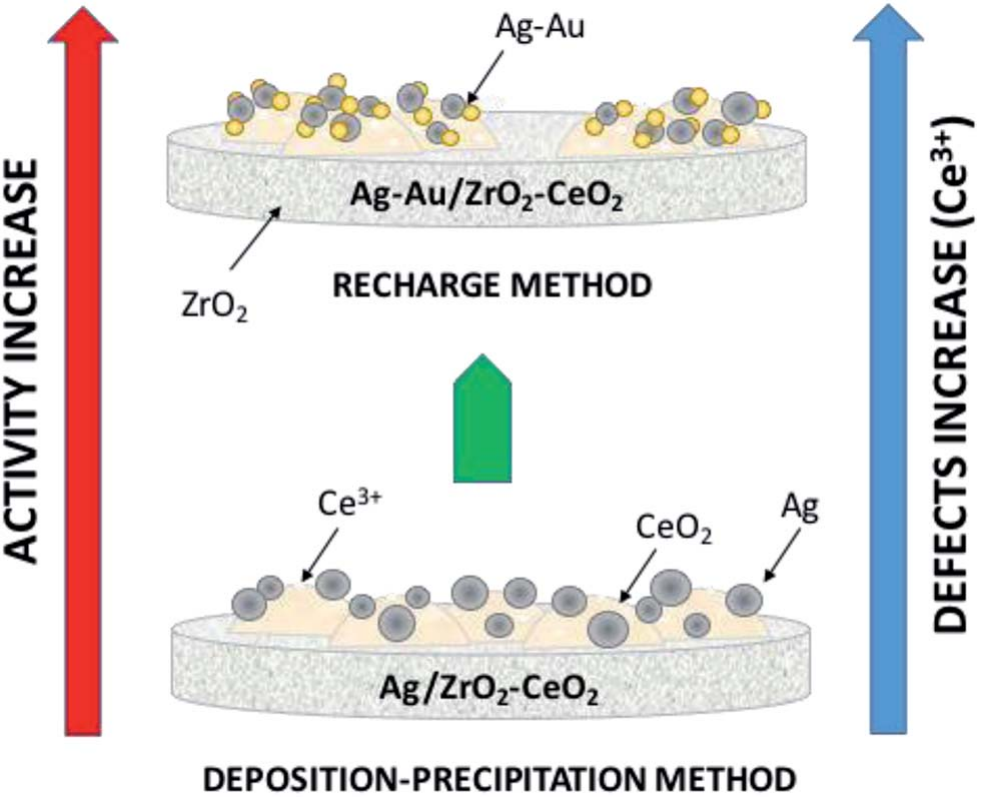
Illustration of the effects involved on catalytic activity on phenol degradation.

CWAO reaction tests showed that the best catalyst was AgAuZrCe20 with 100% conversion in 1 h. Furthermore, the AuAgZrCe20 catalyst obtained a better conversion and selectivity to CO_2_ in comparison with previous results using the Au–Ag/ZrO_2_–CeO_2_ catalyst synthesized by deposition–precipitation precipitation with urea. In this way, the recharge method showed better effects for the synthesis of bimetallic catalyst with gold and silver metals over the mixed oxides ZrO_2_–CeO_2_. The activity may be related to several factors such as: a strong metal support interaction and the reducibility of the support; which influence the release of surface oxygen atoms during the reaction maintaining oxidized silver and gold in metallic state, being a determining factor for the catalytic activity; Fig. 13 illustrates all of the foregoing. Addition of gold changed the properties of silver monometallic catalysts by inhibiting the low formation of intermediates and changed of reaction route by formic acid to CO_2_ and water. In addition, the bimetallic catalyst showed in the reuse cycles the better stability in catalytic wet air oxidation of phenol. These results generate give guidelines to investigate another molar ratio for Ag–Au to improve in the phenol oxidation by CWAO.

## Conflicts of interest

There are no conflicts to declare.

## Supplementary Material
